# Knowledge on CLABSI Prevention Strategies and Associated Factors Among ICU Nurses in Dar es Salaam

**DOI:** 10.24248/eahrj.v9i1.838

**Published:** 2025-09-30

**Authors:** Venance Kahindi, Joel Seme Ambikile, Dickson Mkoka, Neema Mawi, Rashidi Heri

**Affiliations:** a Clinical Nursing Department, Muhimbili University of Health and Allied Sciences (MUHAS), Dar es Salaam, Tanzania; b Nursing Management Department, Muhimbili University of Health and Allied Sciences (MUHAS), Dar es Salaam, Tanzania.

## Abstract

**Background:**

Central line-associated bloodstream infection (CLABSI) is one of the major complications for patients with central lines (CLs) and a leading hospital-acquired infection. This infection is associated with increased length of hospitalization, medical costs, high morbidity, and mortality. As primary caregivers for patients with CLs in the ICU, nurses are crucial in preventing CL-related infections and complications. Despite this key role, there is limited knowledge about ICU nurses’ understanding of CLABSI, especially in low-income countries like Tanzania. Aim: This study aimed to assess knowledge on CLABSI prevention strategies and associated factors among ICU nurses in Dar es Salaam, Tanzania.

**Methods:**

A quantitative cross-sectional analytical study was conducted among 250 ICU nurses in 3 tertiary hospitals. Data were collected from May to July 2023 using a self-administered questionnaire, with CLABSI knowledge assessed using 32 items. SPSS version 25 was employed to analyze data, and logistic regression was performed to identify factors associated with knowledge of CLABSI prevention strategies. In all analyses, statistical significance was set at a *p*-value <.05.

**Results:**

The mean knowledge score was 52.2% (SD = 10.7), with a median of 50%. Among the participants, only 52 (28.2%; 95% CI: 22.6%–33.8%) demonstrated a high level of knowledge regarding CLABSI prevention strategies and care. Receiving in-service CL training (AOR = 5.20; 95% CI: 1.79–15.07; *p* =.002), the availability of CLABSI prevention guidelines (AOR = 2.52; 95% CI: 1.05–6.03; *p*=.038), and the absence of pre-service CL training (AOR = 3.05; 95% CI: 1.33–6.97; *p*=.008) were significantly associated with higher knowledge of CLABSI prevention strategies and care.

**Conclusion:**

ICU nurses demonstrated generally inadequate knowledge of CLABSI prevention strategies, with less than one-third (28.2%) showing a high level of knowledge. In-service CL training, availability of CLABSI guidelines, and absence of pre-service training were significantly associated with higher knowledge, suggesting the strong influence of practical, workplace-based learning. Targeted in-service training and consistent provision of evidence-based guidelines are essential to strengthen ICU nurses’ competence, reduce CLABSI incidence, and improve patient safety.

## BACKGROUND

A central line (CL) is an intravascular catheter that is inserted close to the heart or in one of the major blood vessels. It is used for different purposes, including fluid infusions, withdrawal or transfusion of blood, nutritional support (total parental nutrition), as well as hemodynamic monitoring.^1^ CL is considered a life-saving tool that is commonly used in intensive care units (ICUs) and emergencies for critically ill patients.^23^ Despite its usefulness, if the CL is not properly cared for, it causes serious complications such as deep vein thrombosis, pulmonary embolism, occlusion, dislodgement and infections.^4^ CL-associated bloodstream infections (CLABSIs) are a major and leading complication of the CL, accounting for 89.5% of all healthcare-associated infections (HCAIs) in the ICU.^5^ CLABSIs are associated with prolonged patient hospitalization, increased morbidity and mortality, and financial burden not only to individuals but also to health care systems and health insurances.^6^

In order to prevent the incidence of CLABSIs, several institutions have published clinical practice guidelines for the use of CLs.^7–10^ These guidelines stipulate essential strategies to be implemented, including adhering to hand hygiene requirements, use of aseptic technique, use of maximal sterile barrier precautions, preparation of the insertion site with >0.5% chlorhexidine with alcohol, selection of the best insertion site, and removal of unnecessary CLs. Besides that, healthcare organizations are emphasized to educate, train and conduct regular assessment of knowledge and adherence to guidelines for all healthcare professionals.^11^ Adherence to CLABSI prevention guidelines has been evidenced to reduce both CLABSI rate and its adverse outcomes.^12^

Nurses spend more time with patients in ICUs than other healthcare professionals^13^; thus, they play a vital role in the insertion assistance, care, and maintenance of the CL. However, evidence shows nurses rarely adhere to CLABSI prevention guidelines.^14^ Inadequate knowledge among nurses may hinder adherence to evidence-based guidelines for preventing CLABSI. Previous studies reveal a positive correlation between nurses’ knowledge level and compliance with CLABSI prevention guidelines.^15–17^ Moreover, interventional studies focusing on educating and training nurses on CLABSI prevention guidelines report a significant increase in their knowledge and adherence to evidence-based guidelines practices, which in turn decreases CLABSI rate and its associated burdens^18–20^ Additionally, recent evidence from a systematic review highlight the importance of educating and training healthcare professionals involved in the insertion and care of CLs.^15^

Several studies have been conducted to assess ICU nurses’ knowledge of CLABSI prevention guidelines.^8,10,16,17^ However, to the best of our knowledge, little is known about developing countries, particularly the East African region, where the prevalence of hospital-acquired infection (HAI) is higher (19.7%) compared to other African regions.^18^ An interventional study conducted in Kenya revealed that the CLABSI rates before and after the intervention period were 8.83 and 5.62, respectively.^19^ In Tanzania, a study conducted in a teaching hospital reported that CLABSIs accounted for 43.3% of HCAIs in the medical wards.^20^ Moreover, an unpublished report from Muhimbili National Hospital (MNH) shows that the prevalence of CLABSI in ICUs was 27% (MNH ICU report in 2022). Therefore, this study aimed to assess knowledge on CLABSI prevention strategies and associated factors among ICU nurses in Dar es Salaam, Tanzania. The findings from this study provide insights that can be used to design interventions to increase ICU nurses’ knowledge and thereby reduce the CLABSI rate.

## MATERIALS AND METHODS

### Study Design and Setting

This descriptive cross-sectional study was conducted between May and July 2023 in the Intensive Care Units (ICUs) of three tertiary hospitals in Dar es Salaam: Muhimbili National Hospital (MNH) (both Mloganzila and Upanga campuses), the Jakaya Kikwete Cardiac Institute (JKCI), and the Muhimbili Orthopaedic Institute (MOI). The ICUs were categorized into 4 types based on the type of patients admitted: Medical ICU, Surgical ICU, Pediatric ICU, and Maternity ICU.

MNH Upanga houses five ICUs: Medical, Surgical, Pediatric, and Maternal. This hospital has a capacity of 1500 beds. MNH Mloganzila has a capacity of 609 beds, including Medical and Surgical ICUs. The Jakaya Kikwete Cardiac Institute, with a capacity of 150 beds, includes a Surgical ICU, Pediatric ICU, and Coronary Care Unit. The Muhimbili Orthopedic Institute, serving as a trauma center with a capacity of 340 beds, has one ICU.

These settings were chosen for the study because they are the largest tertiary hospitals in the country, admitting an average of 27 ICU patients per month. Of these, 21% to 80% of patients have a CL used for drug administration, nutrition, hemodynamic monitoring, and hemodialysis. Given this context, it is crucial for nurses working in these critical care settings to possess comprehensive knowledge regarding the care of CLs.

The healthcare system in Tanzania is organized into three levels. The primary level includes dispensaries, health centers, and district hospitals, which provide basic to intermediate care such as preventive, curative, maternity, and minor surgical services, with district hospitals serving as the first referral point. The secondary level consists of regional referral hospitals that offer specialist outpatient and inpatient care, advanced diagnostics, and receive referrals from district hospitals. At the tertiary level are zonal and national referral hospitals, which provide highly specialized and sub-specialized services, advanced diagnostics, as well as teaching and research functions.

### Study Population and Eligibility Criteria

The study population consisted of registered nurses with a diploma or higher education level and at least six months of ICU experience. Nurses who were unavailable during the data collection period, verbally reported feeling unwell, or refused to provide consent were excluded from the study.

### Sampling Procedure and Sample Size

This study recruited nurses working in the ICUs who were available during the data collection period. The sample size was calculated using the single population proportion formula (Cochran's formula)



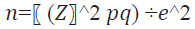



The value of p was obtained from a study conducted in Brazil that assessed nurses’ knowledge on the care of patients with a CL (Latha & Gurung, 2022), where p = 0.82. Accordingly, q = 1 – p = 0.18. Assuming a non-response rate of 10% (i.e., a response rate R = 90%), the adjustment factor is 1/R = 1/0.9 = 1.111. Multiplying this factor by the calculated sample size (n = 226) yields an adjusted sample size of 1.111 × 226 = 250. However, since the total number of nurses active on the duty roster was approximately 300, all available nurses were included, resulting in a response rate of about 75%.

### Data Collection Tool

A self-administered questionnaire with structured questions was used to collect data. The questions were developed based on WHO guidelines for preventing CLABSI and previous literature.^21,22^ The questionnaire comprised two parts: the first part included questions about participants’ socio-demographic details (age, sex, marital status, professional education, working unit, ICU experience, CL training, and the presence of CLABSI guidelines in the working unit); the second part consisted of multiple-choice questions with 32 items assessing knowledge of CLABSI prevention strategies and care. Participants were required to answer all items, with each correct response scored as “1” and each incorrect response scored as “0”. Therefore, the highest possible score was 32, and the lowest 0. The scores were converted to percentages, and those who scored 60% (75^th^ percentile) and above were considered to have high knowledge. The tool was pretested with 13 high-dependence unit (HDU) nurses from the study setting, and any identified ambiguities were addressed.

### Data Collection Procedure

Data collection was conducted from 29^th^ May to 4^th^ July, 2023. Before data collection, all participants were informed about the purpose of the study and their freedom to participate. The researchers distributed questionnaires to the study participants at the beginning of each shift (morning and evening). Due to the nature of the ICU setting, participants were allowed to keep the questionnaire for the entire shift to complete it during their break time. The researchers collected the filled questionnaires from participants at the end of the shift and ensured their completeness.

### Data Analysis

The data were coded, entered, and analyzed using the Statistical Package for the Social Sciences, version 25 (SPSS 25). Before analysis, data cleanliness and completeness were verified to identify any missing data. Mean and standard deviation (±SD) were used to present continuous variables, while frequency and percentage were used for categorical variables. The Shapiro-Wilk test was employed to assess the normality of continuous data. Variables not following a normal distribution were described using median and interquartile ranges (IQR). Bivariate logistic regression analysis was conducted to identify factors associated with knowledge of CLABSI prevention strategies and care. Subsequently, multiple logistic regression analysis was performed, including variables with p-values ≤ 0.2 from the bivariate analysis, as well as factors supported by the literature to be associated with knowledge of CLABSI prevention strategies and care, to further examine these associations. Multicollinearity among the explanatory variables was assessed using the Variance Inflation Factor (VIF), with a VIF value ≤ 2.5 indicating the absence of multicollinearity. The model fit was assessed using the Hosmer–Lemeshow test with a p-value greater than 0.05, indicating a good fit. Statistical significance was set at a p-value < 0.05 for all analyses.

### Ethical Consideration

Ethical clearance for this study was granted by the Research Ethics Committee of Muhimbili University of Health and Allied Sciences (MUHAS) with reference number DA.282/298/01.C/1656. Permission to conduct the study was also obtained from the Executive Directors of Muhimbili National Hospital, Jakaya Kikwete Cardiac Institute, and Muhimbili Orthopedic Institute. Written informed consent was obtained from all participants; they were informed of their right to withdraw from the study at any time, and their participation was entirely voluntary. Confidentiality was strictly maintained by ensuring the anonymity of all documents containing participants’ information.

## RESULTS

### Sociodemographic Characteristics of Participants

A total of 250 participants were included in this study, with a mean age of 34.3 years (SD = 6.7). More than half (59.2%) of the participants were male, and 68.4% were married. The majority were Assistant Nursing Officers (57.6%), and just over half (51.2%) had less than three years of experience working in the ICU. Table 1 summarizes the sociodemographic characteristics of the participants.

**TABLE 1:** Sociodemographic Characteristics of Participants (N= 250)

Variables	Frequency (n)	Percentage (%)
Age in years: Mean (SD)	34.3 (6.7)	
≤34	148	59.2
Above 34	102	40.8
Gender		
Female	148	59.2
Male	102	40.8
Marital status		
Single	79	31.6
Married	171	68.4
Profession education		
Assistant Nurse Officer	144	57.6
Nurse Officer	106	42.4
Working Hospital Unit		
NICU, Pediatric & Maternity ICU	45	18.0
Surgical ICU	107	42.8
Medical ICU	98	39.2
Experience of working as ICU Nurse (months)		
≤3 years and below	128	51.2
Above 3 years	122	48.8
Training on care of CL during Nursing Education		
No	90	36.0
Yes	160	64.0
Received on-job training on CL care		
No	74	29.6
Yes	176	70.4
Presence of CLABSI bundle guideline in the unit		
No	75	30.0
Yes	175	70.0

### Knowledge of CLABSI prevention strategies among ICU nurses and care

The mean knowledge score was 52.2% (SD = 10.7), with a median of 50%. Participants who scored ≥ 60% were classified as having high knowledge of CLABSI prevention strategies, while those who scored below 60% were classified as having low knowledge. Among the participants, only 52 (28.2%) (95% CI: 22.6%, 33.8%) demonstrated high knowledge of CLABSI prevention strategies and care. Table 2 presents the frequency distribution of participants’ knowledge regarding CLABSI prevention strategies. None of the participants answered all questions correctly, with scores ranging from a minimum of 1.6% to a maximum of 94%. Approximately 94% (n = 235) of participants correctly identified how often to assess the central line and the first step when using it. However, only 4 (1.6%) correctly identified the appropriate fluid volume required to flush the central line after use.

**TABLE 2:** Frequency Distribution of Respondent Responses to Knowledge on CLABSI Prevention Strategies and Care

Qn. no.	Assessment questions for CLABSI knowledge and care	Correct responses n (%)
1	How often should the central line be assessed for risks?	235 (94)
2	Before using the central line, what is the first thing to do?	235 (94)
3	How do you check for Central line patency?	196 (78.4)
4.	After using the central line, the healthcare provider should do the following:	102 (40.8)
5	How much fluid volume is needed to flush the catheter lumen after use?	4 (1.6)
6	How often should unused central line lumens be assessed for patency?	73 (29.2)
7	How often do you assess for infection on the central line?	205 (82)
8	How often do you check for the positioning of the central line?	199 (79.6)
9	How often do you wash your hands before using a central line?	198 (79.2)
10	What do you use to wash your hands?	222 (88.8)
11	How often do you wear gloves before using a central line?	233 (93.2)
12	What kind of gloves do you use when you want to dress the central line?	130 (52.0)
13	After how many days do you replace the dressing?	144 (57.6)
14	How many days do you change the transparent drape?	31 (12.4)
15	What is the recommended skin antiseptic preparation?	59 (23.6)
16	What type of dressing drape is proper for central line dressing?	171 (68.4)
17	What do you use to clean a surgical site for disinfection?	37 (14.8)
18	What do you use for disinfection of connectors and injectors?	133 (53.2)
19	How much heparin to administer after using the central line?	166 (66.4)
20	What do you know about CLABSI bundle/strategies?	116 (46.4)
21	What is the key component of infection prevention in a CLABSI bundle?	176 (70.4)
22	When using a central line, maximal sterile barriers are required. This includes?	189 (75.6)
23	What should be done to the patient when inserting a central line?	35 (14.0)
24	What is recommended skin preparation during central line insertion for CLABSI prevention?	61 (24.4)
25	Which is the central line insertion site that is prone to infection?	143 (57.2)
26	Is it recommended to apply an antibiotic ointment at the insertion site of a Central line?	61 (24.4)
27	When should the central line be removed to prevent infection?	13 (5.2)
28	Dressing is immediately replaced when the following happens, except?	91 (36.2)
	What are the signs of infection on the insertion site of the Central Line?	
29	Identified Redness as a sign of central line infection	151 (60.4)
30	Identified swelling as a sign of central line infection	136 (54.4)
31	Identified smelling as a sign of central line infection	82 (32.8)
32	Identified pus discharge as a sign of central line infection	153 (61.2)

### Factors Associated with Knowledge of CLABSI Prevention Strategies and Care

Table 3 presents the results of the bivariate and multiple logistic regression analyses. In the bivariate analysis, having more than three years of work experience (COR = 1.93; 95% CI: 1.06–3.54; *p* <.003), receiving in-service CL training (COR = 3.18; 95% CI: 1.42–7.11; *p*=.002), availability of CLABSI prevention guidelines in the unit (COR = 3.25; 95% CI: 1.45–7.28; *p*<.004), and working in the NICU, Pediatric, or Maternity ICU (COR = 2.96; 95% CI: 1.35–6.50; *p* =.007) were significantly associated with higher knowledge of CLABSI prevention strategies and care.

**TABLE 3:** Factors Associated with Knowledge of CLABSI Prevention Strategies and Care (N=250)

Variables	Low knowledge	High Knowledge	Knowledge of CLABSI prevention strategies & Care			
			Unadjusted COR (95% CI)	*p-value*	Adjusted AOR (95% CI)	*p-value*
Age (years)						
≤34	76 (74.5)	26 (25.5)	Ref.			
Above 34	117 (79.1)	31 (20.9)	1.29 (0.71, 2.34)	.400	0.77 (0.39, 1.59)	.440
Gender						
Male	80 (78.4)	22 (21.6)	Ref.			
Female	113 (76.4)	35 (23.6)	1.12 (0.61, 2.06)	.700	–	–
Marital Status:						
Single	64 (81.0)	15 (19.0)	Ref.			
Married	129 (75.4)	42 (24.6)	1.38 (0.71, 2.69)	.330	–	–
Professional Education						
Assistant Nursing Officers	105 (72.9)	39 (27.1)	1.81 (0.97, 3.39)	.060	1.86 (0.95, 3.65)	.060
Nursing Officers	88 (83.0)	18 (17.0)	Ref			
Work Experience in the ICU						
≤3 Years	106 (82.8)	22 (17.2)	Ref			
>3 years	87 (71.3)	35 (28.7)	1.93 (1.06, 3.54)	.030	1.66 (0.85, 3.25)	.130
Working Hospital Unit				.012	–	–
NICU, Pediatric & Maternity ICU	27 (60.0)	18 (40.0)	2.96 (1.35, 6.50)	.007	–	–
Surgical ICU	86 (80.4)	21 (19.6)	1.08 (0.53, 2.18)	.890	–	–
Medical ICU	80 (81.6)	18 (18.4)	Ref.			
Pre-service CLABSI Training						
Yes	127 (72.2)	49 (27.8)	Ref			
No	66 (89.2)	8 (10.8)	1.15 (0.62, 2.12)	.640	3.05 (1.33, 6.97)	.008
In-service CLABSI Training						
Yes	127 (72.2)	49 (27.8)	3.18 (1.42, 7.11)	.005	5.20 (1.79, 15.07)	.002
No	66 (89.2)	8 (10.8)	Ref			
Presence of CLABSI guidelines						
Yes	126 (72.0)	49 (28.0)	3.25 (1.45, 7.27)	.004	2.52 (1.05, 6.03)	.038
No	67 (89.3)	8 (10.7)	Ref			

Abbreviations: COR = crude odds ratio, AOR = adjusted odds ratio, CI = confidence interval

## DISCUSSION

This study aimed to assess knowledge on CLABSI prevention strategies and associated factors among ICU nurses in Dar es Salaam. The results showed that the nurses had inadequate CLABSI knowledge, with receiving in-service CL training, the availability of CLABSI prevention guidelines, and the absence of pre-service CL training being significantly associated with higher knowledge of CLABSI prevention strategies and care. These results highlight the need to implement targeted in-service training and ensure that CLABSI guidelines are readily accessible within the ICU. These measures are essential for bridging the knowledge gap and improving patient safety. The development and execution of comprehensive educational programs to enhance ICU nurses’ understanding and application of CLABSI prevention protocols are crucial.^23^

Overall, the level of knowledge on CLABSI prevention strategies in this study was low. This implies that ICU nurses in our study had inadequate knowledge regarding CLABSI prevention strategies and care. This finding is consistent with other previous studies conducted in China, Malaysia, and Jordan where low levels of CLABSI knowledge were reported.^8,17,24^ However, our finding contradicts a study conducted in Saudi Arabia, which demonstrated that nurses had an adequate level of knowledge regarding evidence-based CLABSI prevention guidelines.^25^ The difference can be attributed to the presence of local CLABSI guidelines in the Saudi Arabian study, to which the vast majority of participants (84%) referred as their source of information. This highlights the need to take measures to improve ICU nurses’ knowledge on CLABSI, such as on-the-job training and making CLABSI prevention guidelines available. Such educational intervention holds the potential to enhance CLABSI prevention knowledge among ICU nurses.^26^

In our study, receiving in-service CL training was significantly associated with higher knowledge of CLABSI prevention strategies. This suggests that ICU nurses who received on-the-job training had higher knowledge of CLABSI prevention strategies than those who did not. This corroborates with studies conducted in Italy where knowledge was higher in nurses who had received information about the prevention of CLABSIs from formal on-job training such as workshops and courses.^10,27^ Lack of CLABSI prevention training or workshop has been reported in previous studies to be associated with low CLABSI prevention knowledge and is a barrier to the implementation of evidence-based CLABSI prevention guidelines.^16^ Therefore, support for on-the-job training for nurses working in emergency care and ICU needs to be emphasized and should be continuous as supported by evidence.^28,29^ Depending on the circumstances, a simple educational intervention to could be opted for, especially in settings where there is a scarcity of ICU nurses.^30^

Unlike in-service CL training, pre-service CL training in our study was not associated with higher knowledge of CLABSI prevention strategies and care; in fact, it was associated with lower knowledge. This may suggest that exposure to CL topics during pre-service training did not significantly enhance ICU nurses’ knowledge of CLABSI prevention. It is possible that the content or delivery of the pre-service training was inadequate, leading to lower knowledge levels compared to nurses who did not receive such training. Additionally, nurses who were aware of having received pre-service training may have made less effort to update or improve their knowledge and skills, assuming their prior training was sufficient. These findings are contrary to a study in Saudi Arabia where nurses who had educational courses on central vascular catheter (CVC) maintenance had higher knowledge.^31^ To improve CL or CVC knowledge among nursing students, a structured educational program focusing on theoretical and clinical competencies is recommended.^32^ The reason for pre-service training not predicting CLABSI knowledge in our study may be attributed to the quality and relevance of the content, as it might have focused on theoretical knowledge rather than practical skills, thus not providing opportunities for hands-on practice.^33^

In the current study, the availability of the CLABSI prevention guideline predicted higher knowledge on CLABSI prevention strategies. This implies that having access to the CLABSI prevention guideline helped ICU nurses to learn more about CLABSI prevention strategies. This is supported by a study conducted in Italy, where knowledge about evidence-based practices for preventing CLABSI was significantly higher among nurses working in hospitals that had a written policy about CVC maintenance for the prevention of CLABSI.^27^ Shortage of necessary equipment is one of the most critical implementation barriers of evidence-based guidelines to the prevention of CLABSI.^16^ This stresses the need for health facility administrators to ensure that guidelines for CLABSI prevention are made available in key settings such as ICUs, which the evidence shows should be standardized.^29^

While age, gender, marital status, and experience of working in the ICU did not show statistical significance with knowledge of CLABSI prevention strategies among ICU nurses in our study, other studies have shown varying relationships. A study in Jordan showed that age was the single predictor of knowledge of CLABSI prevention.^24^ In another study in Poland, age and length of service had no effect on nurses’ knowledge of CLABSI.^29^ Yet in another study conducted in Saudi Arabia, higher CLABSI prevention knowledge levels were significantly associated with older age and longer ICU nursing experience.^31^ As previously discussed, several factors can account for the differences observed in the impact of sociodemographic factors on CLABSI knowledge across various studies. These include methodological variations, such as differences in sample size, as well as participant characteristics like educational background, years of experience, and workplace environment.

There are some potential limitations that need to be considered in this study. First, the cross-sectional nature of this study does not allow for determining the direction of influence between the assessed variables. We aimed to merely assess factors associated with CLABSI prevention knowledge among ICU nurses. Second, this study was conducted in tertiary hospitals, which raises some concerns about generalizability and comparability. Being the highest level of health facilities, tertiary hospitals are different from lower-level health facilities since they are more equipped with facilities and employ nurses with a higher level of education and specialities. Despite these limitations, this study provides invaluable insights into knowledge of CLABSI prevention strategies among ICU nurses and the associated factors.

In conclusion, this study revealed that ICU nurses had generally inadequate knowledge of CLABSI prevention strategies, with less than one-third demonstrating a high level of knowledge. Importantly, in-service CL training, the availability of CLABSI prevention guidelines, and the absence of CL pre-service training were significantly associated with higher knowledge, possibly reflecting the greater impact of practical, workplace-based learning. These findings highlight the urgent need for targeted, ongoing in-service training programs and the consistent provision of accessible, evidence-based CLABSI prevention guidelines in ICU settings. Hospital administrators and policymakers should prioritise resource allocation, structured training curricula, and regular competency assessments to ensure that ICU nurses are equipped with the necessary skills and knowledge to prevent CLABSI. Such measures are critical for improving adherence to best practices, reducing preventable infections, enhancing patient safety, and ultimately improving ICU patient outcomes.
